# Hospital Meetings

**Published:** 1894-10-06

**Authors:** 


### HOSPITAL MEETINGS.

### CHARING CROSS MEDICAL SCHOOL.

The members of the teaching staff at Charing Cross have hit upon a method of observing the first day of the winter session which is well fitted to establish closer relations between the school and its friends. On the invitation of the staff a large number of ladies and gentlemen attended a conversazione in the medical school buildings on Monday evening. Prettily decorated, museum, lecture-room, and laboratory wore an unwonted aspect of gaiety, and there were pictures, chemical and physiological apparatus, microscopic slides, and other objects of interest for visitors to look at. At an early stage in the evening the prizes won last session were distributed by Professor Macalister, of Cambridge in the presence of a large company of students and their friends. Mr. Stanley Boyd, Dean of the school, presided, and Professor Macalister gave a brief and suitable address. The arrangements made for the entertainment of the guests included a lantern lecture by Dr. Abercrombie, subject, "A Tour in Greece," a concert, and a "new and original musical dialogue," written by Mr. George Hawtrey—a bright, clever piece which was well received. Altogether it was a very enjoyable evening, and the hospitality of the medical school staff was highly appreciated.

